# Spike-timing control by dendritic plateau potentials in the presence of synaptic barrages

**DOI:** 10.3389/fncom.2014.00089

**Published:** 2014-08-14

**Authors:** Adam S. Shai, Christof Koch, Costas A. Anastassiou

**Affiliations:** ^1^Division of Biology and Bioengineering, California Institute of TechnologyPasadena, CA, USA; ^2^Allen Institute for Brain ScienceSeattle, WA, USA

**Keywords:** plateau potentials, dendrites, spike timing, inhibition, non-linear dendrites

## Abstract

Apical and tuft dendrites of pyramidal neurons support regenerative electrical potentials, giving rise to long-lasting (approximately hundreds of milliseconds) and strong (~50 mV from rest) depolarizations. Such plateau events rely on clustered glutamatergic input, can be mediated by calcium or by NMDA currents, and often generate somatic depolarizations that last for the time course of the dendritic plateau event. We address the computational significance of such single-neuron processing via reduced but biophysically realistic modeling. We introduce a model based on two discrete integration zones, a somatic and a dendritic one, that communicate from the dendritic to the somatic compartment via a long plateau-conductance. We show principled differences in the way dendritic vs. somatic inhibition controls spike timing, and demonstrate how this could implement spike time control in the face of barrages of synaptic inputs.

## Introduction

It has been established that multiple non-linear mechanisms exist in the dendrites of excitatory neurons, such as layer 5 cortical pyramidal neurons. In particular, long-lasting plateau potentials far from the soma in the apical, basal, and tuft dendrites, are supported by voltage-gated calcium (Ca) and sodium channels, as well as NMDA receptors (Magee et al., 1998; Larkum et al., 1999, 2007). Importantly, glutamate uncaging experiments have shown a strong correlation between non-linear dendritic events and long-lasting somatic depolarizations on the order of hundreds of milliseconds and 10–25 mV (Kamondi et al., 1998; Antic et al., 2010). Furthermore, genetically distinct groups of interneurons differentially target perisomatic and dendritic tuft regions of single pyramidal cells in regions like the hippocampus (Freund and Buzsáki, 1996; Royer et al., 2012) and neocortex (Ascoli et al., 2008). Recent *in vitro* and *in vivo* work demonstrates that dendritic inhibition can play strikingly different roles than perisomatic inhibition with respect to action potential spiking output (Palmer et al., 2012; Royer et al., 2012). In addition, computational studies have shown that spatial distributions of inhibitory input can independently affect somatic and dendritic regions such that the effect of plateau potentials on the soma is reduced without directly changing the properties of the dendritic plateau potential itself (Gidon and Segev, 2012; Jadi et al., 2012).

How can single cells use such a biophysical setup supporting dendritic plateau potentials with spatial distributions of both intrinsic conductances and synaptic inputs to control action potential output? To study mechanisms of spike timing in pyramidal neurons we compare single neuron processing in a conventional leaky integrate-and-fire (LIF) unit, a two compartment biophysical model with 6 Hodgkin-Huxley-like currents, and a novel abstracted two-stage LIF model taking into account the relevant aspects of dendritic electrogenesis found in pyramidal neurons. In particular, we study the impact of long-lasting dendritic depolarization on somatic spiking with and without somatic inhibition. We show that a simple 2-stage LIF model, like the more complicated compartmental model it abstracts, gives rise to precise spike timing in the presence of barrages of excitatory and inhibitory inputs. Important to this mechanistic hypothesis are the distinct effects inputs into the dendritic and perisomatic regions produce. We further demonstrate how our model explains recent experimental results in hippocampal place cells where a decrease of dendritic inhibition causes a decrease in phase precession and enforces spiking around a single phase.

## Materials and methods

### Synaptic inputs into the models

**Figure 1:**
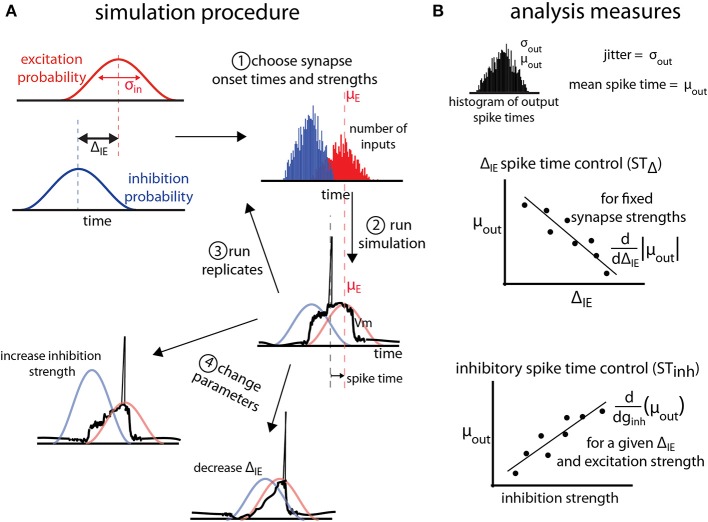
**General protocol for simulations. (A)** Excitatory (red) and inhibitory (blue) synaptic barrages impinge on single neuron models according to temporal probability distributions with standard deviation σ_in_. Excitatory and inhibitory input barrages are temporally offset by the temporal input offset, Δ_IE_. After (1) choosing onset times and synaptic strengths for the inputs, (2) simulations are conducted and the spike time (measured relative to the mean of the excitatory probability distribution, and, here, negative) is recorded. For each parameter triplet of excitatory and inhibitory strengths and Δ_IE_, (3) replicates are run. (4) Parameter space is explored by choosing new parameter triplets and running new simulations. **(B)** Analysis of the simulations is performed on the output distribution of the spike times over all replicates for a given parameter set. The jitter and mean spike time are defined as the standard deviation and mean of the output spike times. To measure the control of spike timing by Δ_IE_ (ST_Δ_) and inhibition strength (ST_inh_), we find the absolute value of the slope of the best linear fit of the mean spike time as a function of Δ_IE_ or inhibition strength (*g*_inh_). These values are thus measures of how much mean spike times shift as the temporal input offset or inhibition strength changes.

This study features three different single cell models: leaky-integrate-and-fire (1LIF), conventional two compartmental, and a two-component leaky-integrate-and-fire (2LIF, see below, model available for download at https://senselab.med.yale.edu/modeldb/ upon publication). In all models excitatory and inhibitory inputs are implemented as conductance increases described via alpha functions with reversal potentials of 65 and −10 mV relative to rest, respectively. Both synaptic types impinge on the membrane with conductance profiles described by

$$g(t)=g_{\text{max}}\left(\frac{t-t_{0}}{\tau }\right)e^{-\left(t-t_{0}-\tau \right)/\tau }$$

where *g*_max_ is the maximum conductance of a single synapse, *t*_0_ is the synapse onset time, and τ is the synapse time constant. A *barrage* of postsynaptic events is defined as a group of *n* events sharing the same *g*_max_, τ, *V_rev_* (reversal potential), and a vector of *t*_0_s of length *n* chosen from the same temporal probability distribution. A normal probability distribution with mean time μ_in_ and standard deviation σ_in_ generates a *t*_0_ for each of the *n* synaptic events in each barrage (Figure 1A). Because spike time is defined as the time at which the membrane potential first crosses threshold relative to μ_exc_ (the mean time μ of the excitatory barrage probability density function), voltage resetting does not affect the analysis and is not included in the simulations.

To compare synapse strengths between different models, the effect of barrages on membrane voltage needs to be normalized. To do so, we divide the synapse strengths (maximum conductance, given in nS) by the threshold synapse strength where an excitatory barrage in the absence of an inhibitory barrage causes a spike. In the 1LIF and 2LIF models this is at 0.97 nS, and in the modified Mainen-Sejnowski 2-compartment model this is found to be 0.078 nS [the original Mainen-Sejnowski model has a high input resistance, eliciting spikes with a 10 pA DC current injection into the somatic compartment (Mainen and Sejnowski, 1996), about an order of magnitude lower than in experiment]. Hence, for all simulations, the parameter space searched was from 1 to 2 times threshold strength for excitation, and from 0 to 5 times that strength for inhibition.

Parameters for excitatory barrages are σ_in_ = 40 ms, number of synaptic events per barrage *n* = 100, τ = 0.5 ms, and *g*_max_ ranging from 1.0 to 2.0 nS. Parameters for inhibitory barrages are σ = 40 ms, number of synaptic events per barrage = 200, τ = 0.75 ms, and *g*_max_ ranging from 0 to 5 nS. These parameters are comparable to a previous modeling study on the number of synaptic inputs needed to elicit long-lasting regenerative potentials in the tuft dendrites of L5 pyramidal neurons (Larkum et al., 2009). Every simulation starts with neurons completely at rest. The *temporal input offset* is defined as the temporal difference μ_inh_− μ_exc_ (Figure 1A). The *input jitter*, σ_in_, is defined as the standard deviation of the excitatory barrage probability distribution and has units of milliseconds. Since the standard deviation of any input distribution, σ, is a defined constant unit of time (in this study 40 ms), all time measurements can be expressed in units of σ_in_ (so that 0.5 σ_in_ would be equivalent to 20 ms and 2.0 σ_in_ would be equivalent to 80 ms). The *spike time* is the time of the first voltage threshold crossing relative to μ_exc_, and is measured in units of σ_in_. The *jitter*, σ_out_, is defined as the standard deviation of the output *spike times* (Figure 1B) (Marsalek et al., 1997), and is also reported in units of σ_in_. Simulations in the 1LIF and 2LIF are conducted through a range of 6 input offsets, from 0 to 2 σ_in_, 50 excitatory synapse strengths, and 50 inhibitory synapse strengths, and for 25 excitatory and inhibitory synapse strengths for the 2-compartment model. For each parameter triplet, 1000 replicates are conducted in the 1LIF and 2LIF cases (making a total of 15,000,000 simulations), and 20 replicates in the 2-compartment model (due to the increased computational time), each with new input times chosen from their temporal probability distributions. Only those experiments where excitation is strong enough to allow for at least half of the replicates to cross spiking threshold are used.

To quantify the ability of the temporal input offset to influence spike timing, we use a measure we term *shift in spike time due to temporal input offset* (*ST*_Δ_). *ST*_Δ_ is the rate at which changing the temporal input offset changes the mean spike time for fixed excitatory and inhibitory strengths. *ST*_Δ_ is calculated by finding the absolute value of the slope of the least squares fit line to the mean spike times as a function of Δ_IE_ for a given excitation and inhibition strength. In other words, *ST*_Δ_ reports how many σ_in_ the spike time changes by changing the input offset by 1 σ_in_, and is thus measured in units of σ_in_/σ_in_. The absolute value is taken in order to make *ST*_Δ_ a direct measure of spike-time control (in this study, all values of *ST*_Δ_ would be negative if the absolute value was not taken, see Figure 1B). Importantly, in all 1LIF simulations, the 0 σ_in_ input offset cases produce mean spike times that do not follow the linear trend of the other input offsets (see Supplemental Materials). Thus, including the 0 σ_in_ input offset data in the *ST*_Δ_ calculations would underestimate the ability of input offset to influence spike timing, and so were not included.

To quantify the ability of inhibitory barrages to influence spike timing, we use a measure we term the *shift in spike time due to inhibition strength* (*ST*_inh_). *ST*_inh_ is the rate at which the mean spike time changes with changing inhibition strength, for a given excitatory strength. In other words, *ST*_inh_ is a measure of how many σ_in_ the spike time changes by changing the inhibition strength by 1 nS, and is thus given in units of σ/nS. *ST*_inh_ is calculated by finding the slope of the least squares linear fit to mean spike time as a function of inhibition strength, for a given excitation strength.

### A single leaky-integrate-and-fire unit (1LIF)

A single compartment leaky-integrate-and-fire neuron (1LIF) is implemented in *Matlab* (The MathWorks Inc., Natick, MA) using the method of finite differences on the dynamic equation $c_{m}\frac{dV(t)}{dt}=\sum_{i}g_{i}(t)\left[V(t)-V_{i,rev}\right]-\frac{V(t)}{r_{m}}$, where *c_m_*, *g_i_*, *r_m_*, *V_i,rev_*, and *V* are the membrane capacitance, conductance change of synapse *i*, membrane resistance, reversal potential of synapse *i*, and the membrane potential, respectively. Simulations exploring how input offset between excitatory and inhibitory barrages affect spike jitter and the dynamic range of spiking use 1LIF with the following parameters, treating the 1LIF as an isopotential sphere (Koch, 1999): *r_m_* = 80 MΩ, *c_m_* = 13 pF (assuming a radius of 10 μm this is equivalent to 1.035 μF·cm^−2^), *V*_thresh_ = 16 mV, and *V*_rest_ = 0 mV.

### A two-compartment Hodgkin-Huxley model supporting dendritic plateau events

A two-compartment model of a pyramidal neuron previously created (Mainen and Sejnowski, 1996) to study the effect of dendrites on action potential output is modified to account for dendritic spiking. The original model features a somatic compartment with sodium (Na) and potassium (K_v_) Hodgkin-Huxley style currents to support action-potential spiking, and a dendritic compartment with Na, muscarinic potassium (K_m_), calcium-dependent potassium (K_Ca_), and high-voltage activated calcium (Ca_HVA_) currents to support dendritic processing. The dendritic compartment additionally contains a linear resistance and capacitance, and the two compartments are joined with a linear resistance (**Figure 3A**). In pyramidal neurons, dendritic calcium electrogenesis has been shown to be dependent on both high and low-threshold calcium currents (Markram and Sakmann, 1994; Perez-Garci et al., 2013). A low-voltage activated calcium Hodgkin-Huxley current is thus added to the dendritic compartment. The current has the following parameters, based off the Ca_LVA_ mechanism used in Hay et al. (2011): m_∞_ = 1/(1+exp(−(V+40)/6)); h_∞_ = 1/(1+exp((V+90)/6.4)); τ_m_ = 5 + 20/(1+exp((V+35)/5)); τ_h_ = 75 + 50/(1+exp((V+50)/7)); *N*_m_ = 2; *N*_h_ = 1. This model is implemented in the NEURON programming environment (Carnevale and Hines, 2006).

### A two-component LIF model (2LIF)

A two-component leaky-integrate-and-fire (2LIF) model is implemented using two coupled 1LIFs (**Figure 3A**). We refer to one compartment as the *dendritic* and the other as the *somatic compartment*. These compartments interact via a single conductance activated in the somatic-unit whenever the dendritic-unit reaches threshold (here set to 16 mV above rest). This conductance, referred to as the *plateau-conductance*, is a constant conductance lasting 3σ_in_ (120 ms) of strength *g*_plat_ which, in the absence of any somatic input, gives rise to a *V*_thresh_ + 1 mV constant depolarization in the somatic compartment. That is, in the absence of inhibition, the dendritic plateau potential will trigger a somatic spike, as seen with calcium events induced in the dendrites of pyramidal neurons (Larkum et al., 2009; Antic et al., 2010).

### Comparisons of temporal offset spike time control in 1LIF and 2LIF models

In the 2LIF model, barrages of input impinge onto the dendritic and somatic compartments independently. Inhibitory barrages impinging on the dendritic compartment are named *direct barrages*, since they directly affect the timing of the plateau conductance. Inhibitory barrages into the somatic compartment are named *gating barrages*, since they gate the effect of the plateau conductance on somatic spiking. Note that unlike a conventional two-compartment model, the somatic membrane potential does not influence the dendritic membrane potential.

*Spike* and *dendritic plateau timing* are defined as the time of the first threshold crossing in the somatic and dendritic-compartment, respectively, relative to the mean of the dendritic excitatory barrage probability density function.

**Figure 2:**
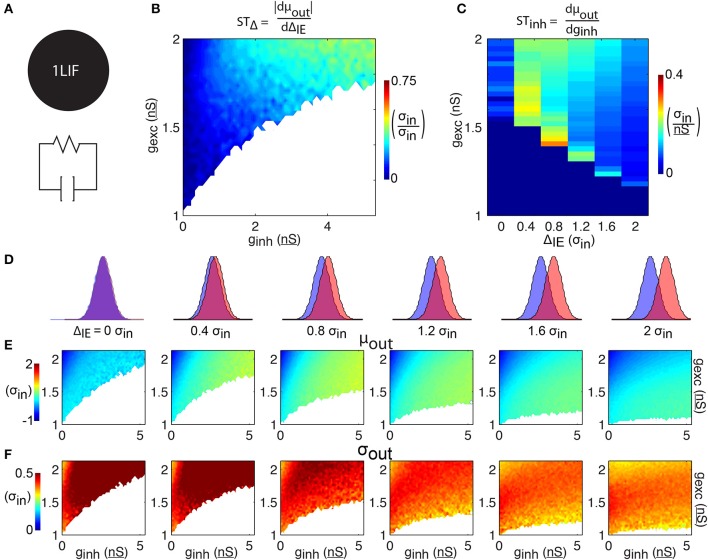
**1LIF simulations. (A)** The leaky-integrate-and-fire unit (1LIF) is a single compartment with a leak conductance and membrane capacitance. **(B)** The shift in spike time due to the temporal input offset is plotted as a function of excitatory and inhibitory synapse strength (*g*_exc_ and *g*_inh_). White areas occur due to the inability of the 1LIF to spike when excitation is not strong enough to elicit a spike given a certain amount of inhibition. **(C)** The shift in spike time due to the inhibitory strength is plotted as a function of temporal input offset and excitatory synapse strength. For each of 6 input offsets tested (columns in **D**) mean spike time **(E)** and output jitter **(F)** are plotted.

In the analysis of spike time control in the 2LIF model (**Figure 4**), excitatory barrages impinge onto the dendritic compartment, and inhibitory barrages impinge on the somatic one. The same parameters for the barrages in the 1LIF analysis (Figure 2) are used in this analysis. In the comparison of gating and direct inhibition in the 2LIF model (**Figure 5**), excitatory barrages have *g*_max_ = 1.2 nS and inhibitory barrages have *g*_max_ = 5 nS. 1000 permutations are carried out at each of 108 input offsets spanning linearly from 0 to 2.5 σ_in_.

## Results

### Simulations and analysis exploring mechanisms of spike timing in single cells

How might spatially distributed conductances such as those supporting plateau potentials interact with spatio-temporally distributed inhibitory inputs to give rise to spike time control? In order to establish a method for comparison, we first explain the general simulation and analysis framework used in this work.

In a single cell model, excitatory, and inhibitory barrages arrive following σ_in_ = 40 ms probability density functions (Figure 1A, for details see Materials and Methods). We focus on a 40 ms input jitter due to experimental measurement of the distribution of presynaptic cell firing in entorhinal cortex that projects to the hippocampus (Mizuseki et al., 2009). Excitatory and inhibitory barrages arrive with a *temporal input offset* (Δ_IE_; Figure 1A). For each simulation, excitatory and inhibitory synaptic strengths (*g*_inh_ in Figure 1B) and Δ_IE_ are chosen. Synaptic strengths are given in units of nano-Siemens normalized to the excitation threshold for spiking (denoted nS, see Materials and Methods). The output of the simulation is the spike time, measured relative to mean of the excitatory barrage probability density function (Figure 1A2), and is thus a negative number if the spike precedes or a positive number if the spike follows the mean time of the excitatory barrage. In order to make the relation of spike output statistics to synaptic input statistics explicit, we analyze spike times in units of σ_in_ (e.g., an output spike time of −0.5σ_in_ refers to a spike that occurs 20 ms before the mean of the σ_in_ = 40 ms excitatory synaptic barrage). Multiple simulation replicates are performed for each parameter set, and the parameter space is explored (see Materials and Methods). For a given parameter set, *jitter* (σ_out_) and *mean spike time* (μ_out_) are defined as the standard deviation and mean of the spike times over all replicates (Figure 1B, top).

Control of spike time is measured in two ways. First, the *shift in spike timing due to temporal input offset* (ST_Δ_) is the slope of the best-fit line of μ_out_ as a function of Δ_IE_, and thus estimates the shift in mean spike time by a unit increase in Δ_IE_ (Figure 1B, middle). Second, the *shift in spike timing due to inhibition strength* (ST_Inh_), is the slope of the best-fit line of μ_out_ as a function of the inhibitory barrage synapse strength (*g*_inh_), and thus estimates the shift in spike timing by changes in the balance between excitation and inhibition.

### Spike time control in a single leaky-integrate-and-fire unit

To establish a baseline for comparison, we begin by quantifying the relationship between barrages of synaptic events and spike timing in a single leaky-integrate-and-fire unit (1LIF, Figure 2A) with no spatial extent of membrane conductances or synapses. In particular, we are interested in the control of spike timing by changes in Δ_IE_ and inhibition strength (Figures 2B,C).

For each of six values of Δ_IE_ we test (Figure 2D), increasing excitation strength shifts spiking to earlier times, while increasing inhibition strength delays spiking (Figure 2E). In general, jitter decreases as Δ_IE_ increases (Figure 2F). Although spike timing is generally earlier as Δ_IE_ increases (however the 0 Δ_IE_ case does not follow this trend), the *shift in spike timing due to* Δ_IE_ (*ST*_Δ_, i.e., how many σ_in_ later the spike occurs given an increase in input offset of 1 σ_in_) is weak, with only 30.6% of the parameter space able to change spike timing by more than 0.25 σ_in_ with a 1 σ_in_ increase in Δ_IE_ (Figure 2B). In general, increasing the strength of inhibition increases *ST*_Δ_ (Figure 2B).

Spike time also varies as a function of the strengths of the inhibitory inputs. When excitatory and inhibitory distributions have no temporal input offset (i.e., Δ_IE_ = 0 and their input distributions are the same; Figures 2D–F, 1st column), the *shift in spike timing due to inhibition strength* (*ST*_Inh_, how many σ_in_ later the spike occurs given a 1 nS increase in inhibition strength) reaches a peak of 0.1 σ_in_/nS and an average jitter of 0.9 σ_in_ (Figure 2C). The no offset condition thus represents an especially poor case of spike time control compared to cases with barrages separated in time, as it has both low *ST*_inh_ (Figure 2C) and high jitter (Figure 2F). For every other Δ_IE_, *ST*_inh_ is higher, and decreases as Δ_IE_ increases.

Importantly, the decrease in jitter induced by increasing Δ_IE_ (Figure 2F) comes with the tradeoff that ST_inh_ is reduced. For example, we find that simulations with Δ_IE_ of 2.0 σ_in_ have ST_Inh_ reaching 0.1 σ_in_/nS, while simulations with Δ_IE_ of 0.4 σ_in_ reach 0.3 σ_in_/nS (Figure 2C). Although the latter simulations have relatively high ST_Inh_, they also have higher jitter, reaching σ_out_ of 0.8 σ_in_ for 0.4 σ_in_ input offset, whereas cases with 2.0 σ_in_ input offset have a maximum σ_out_ of 0.5 σ_in_ (Figure 2C). Similarly, shifts in mean spike time due to increasing Δ_IE_ (Figure 2E, from left to right) do not sustain constant levels of jitter (Figure 2F, from left to right) over the range Δ_IE_ where spike timing is shifted.

### Increased spike time control achieved with a plateau potential mechanism

**Figure 3:**
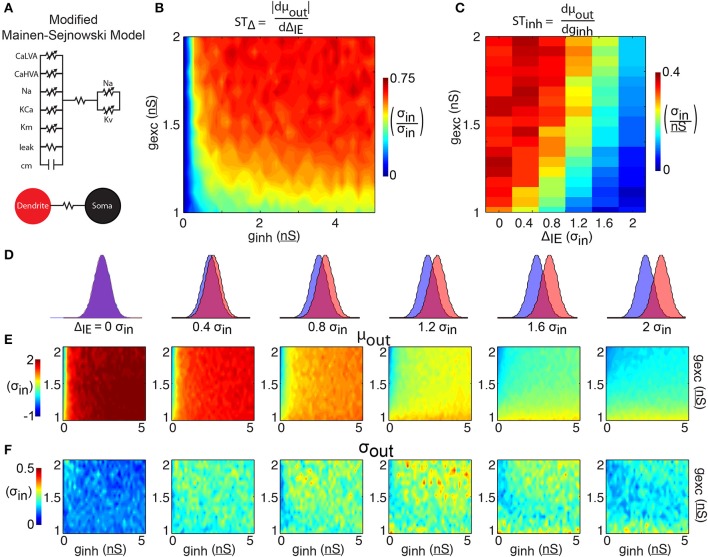
**2-compartment model. (A)** The 2-compartment model features 6 Hodgkin-Huxley conductances separated into a somatic and dendritic compartment. The two compartments communicate via a linear resistance. **(B)** The shift in spike time due to the temporal input offset is plotted as a function of excitatory and inhibitory synapse strength (*g*_exc_ and *g*_inh_). **(C)** The shift in spike time due to the inhibitory strength is plotted as a function of temporal input offset and excitatory synapse strength. For each of 6 input offsets tested (columns in **D**) mean spike time **(E)** and output jitter **(F)** are plotted.

Recent experimental results implicate inhibition in the apical and tuft dendrites of pyramidal neurons in the control of spike timing (Royer et al., 2012). Interestingly, this spatially restricted input corresponds to a region in pyramidal neurons known to have a high density of L-type Ca^2+^ channels (Perez-Garci et al., 2013). This “Ca-hotzone” supports a second spiking zone where depolarizations can cause a long-lasting pleateau event (Larkum et al., 2009). To address the potential role of the Ca-hotzone and the associated plateau potentials, we adopt a two-compartment Mainen-Sejnowski pyramidal neuron, originally created to explore the role of the apical dendrites on neural output (Mainen and Sejnowski, 1996). We add an additional Hodgkin-Huxley type low-voltage activated Ca channel to the dendritic compartment to support the regenerative Ca-spike known to occur in the apical dendrites (Figure 3A).

The results of simulations with this two-compartment model (Figure 3) show both increases in spike time control and decreases in jitter compared to the 1LIF simulations (Figure 2). Although the 1LIF also showed earlier spiking as Δ_IE_ increased, here the changes in mean spike time are greater and more sustained across the range of Δ_IE_ tested (Figure 3E, left to right), thus ST_Δ_ is greater in most of the parameter space (Figure 3B). A similarity with the 1LIF is observed in the decreasing ability for inhibition strength to shift spike timing as Δ_IE_ increases (Figure 3C). However, in the two-compartment model greater levels of ST_inh_ are found, marking a greater ability to control spike timing by changing either Δ_IE_ or the relative strengths of excitatory and inhibitory input. Importantly, spike time jitter decreases in the entire parameter space compared to 1LIF simulations. This suggests that separating excitatory and inhibitory inputs into separate electrophysiological regions can be a mechanism for single neurons to control spike output and more effectively decrease jitter.

### Control of spike timing in a two-component LIF model

**Figure 4:**
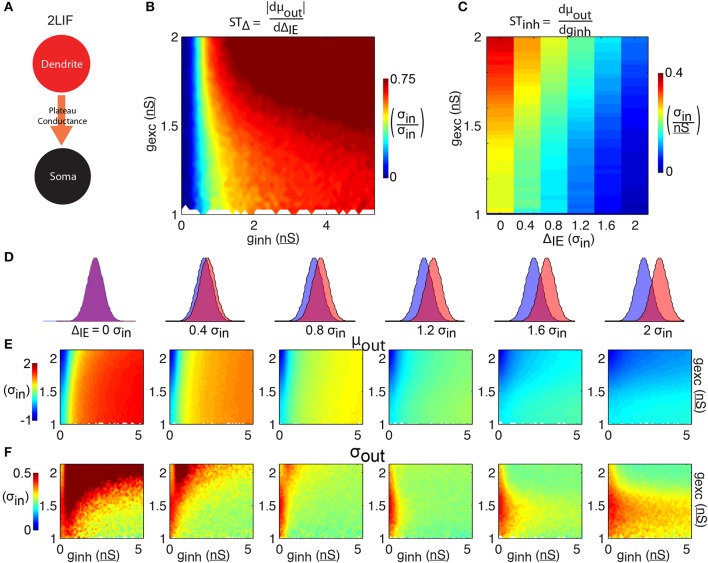
**2LIF model. (A)** The 2LIF consist of 2 leaky-integrate-and-fire units communicating via a long-lasting plateau-conductance. Whenever the dendritic-unit reaches threshold, a plateau-conductance opens in the somatic-unit. **(B)** The shift in spike time due to the temporal input offset is plotted as a function of excitatory and inhibitory synapse strength (*g*_exc_ and *g*_inh_). **(C)** The shift in spike time due to the inhibitory strength is plotted as a function of temporal input offset and excitatory synapse strength. For each of 6 input offsets tested (columns in **D**) mean spike time **(E)** and output jitter **(F)** are plotted.

To study the essential components of the single-cell spike time control mechanism explored in the 2-compartment model, we use a 2-compartmental LIF abstraction for a single pyramidal cell (2LIF, see Materials and Methods; Figure 4A). This model has the additional advantage of a structure more closely related to the 1LIF, thus comparison with the 2LIF is more direct than with the two-compartment model.

The 2LIF consists of two LIF units that act independently except when the dendritic-unit reaches threshold, which activates a long-lasting constant plateau-conductance in the somatic-unit (Figure 4A, see Materials and Methods). As before, increasing inhibition strength delays spiking (Figure 4E). Compared to the 1LIF case, increasing excitation has little effect on spike timing (Figure 4E), due to the stereotyped amplitude of the plateau-conductance. Importantly, across all Δ_IE_ we observe a decrease in jitter compared to the 1LIF case (Figure 4F). Additionally, as Δ_IE_ increases, mean spike time decreases rapidly compared to the 1LIF case. ST_Δ_ is thus greater than 0.6 σ_in_/σ_in_ for the majority of the parameter space tested (Figure 4B), and greater than the 1LIF ST_Δ_ values for 95.9% of parameter space tested (comparing Figure 4B to Figure 2B). Here, the ST_Δ_ values are greater than 0.25 σ_in_/σ_in_ for 87.9% of the parameter space tested, compared to 30.6% for the 1LIF simulations. Additionally, in all the parameter space tested in which the somatic inhibitory strength is less than or equal to 0.5 nS, 88.0% of the simulations had an ST_Δ_ less than 0.25 σ_in_/σ_in_ (Figure 4B), whereas only 0.5% of the rest of parameter space tested has such an ST_Δ_. Thus, somatic inhibition is required for robust spike timing control, and the weakening of somatic inhibition is detrimental for such control.

ST_inh_ decreases as Δ_IE_ increases (Figure 4C). Unlike the 1LIF case, this modulation of ST_inh_ by changing Δ_IE_ is not generally accompanied by large increases in jitter, except if there is no offset between excitation and inhibition. For Δ_IE_ of 0.4 σ_in_ or greater, jitter averaged over a given excitation strength remain less than 0.5 σ_in_ in all cases (Figure 4F).

### Comparing mechanisms of spike time control

**Figure 5:**
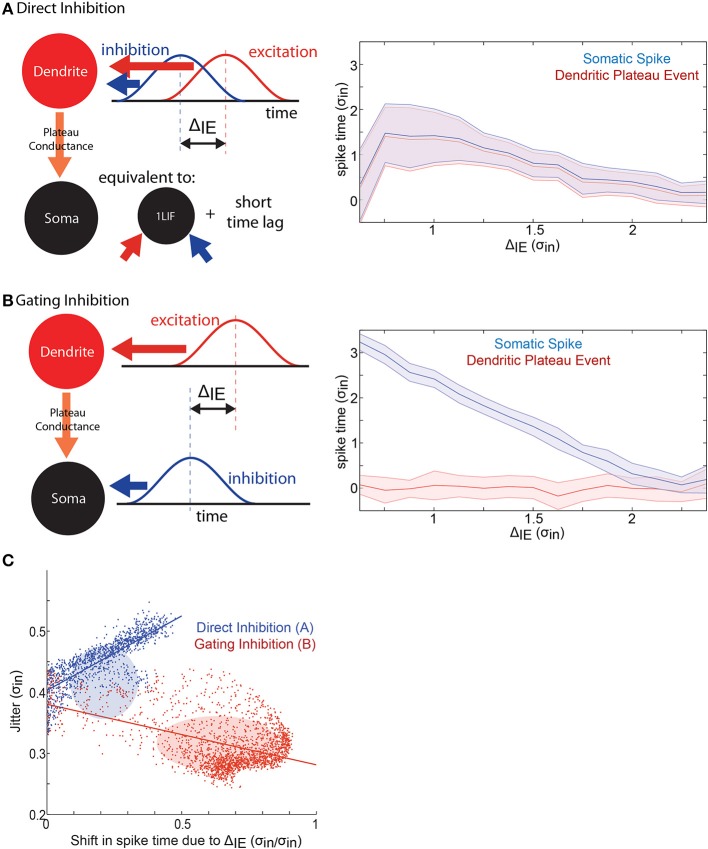
**A mechanism for spike time control. (A)** In the direct inhibition case, excitation and inhibition both impinge on the dendritic-unit. This is equivalent to a 1LIF with a short time lag between threshold crossing and spiking. Here, 1000 replications were conducted at each of 108 different input offsets. (right) Plot of the time of the plateau potential (relative to the mean of the synaptic excitatory barrage) (red), and the somatic spike time (blue), as a function of the temporal input offset between the mean timing of the excitatory and the inhibitory barrages. Output jitter is shown by the shaded area. **(B)** In the gating inhibition case, excitation impinges on the dendritic-unit while inhibition impinges on the somatic-unit where it interacts with the plateau-conductance. (Right) Same as **(A)** for the gating inhibition case. The timing of the plateau event does not depend on the input offset, while the timing of the somatic spike decreases with increasing offset between excitation and inhibition. Note that the spatially separated inputs give rise to steady low-jitter spike time modulation compared to the direct inhibition case. **(C)** Scatter plots of every data point tested in the 1LIF (Figure 2) and 2LIF (Figure 4) showing how jitter varies as a function of offset spike time control. Ellipses show center of mass with height and width of ±1 standard deviation. Lines show linear best fits.

Finally, we compare the effect of dendritic (“direct,” Figure 5A) and somatic (“gating,” Figure 5B) inhibitory barrages on *ST*_Δ_. Importantly, the direct inhibition 2LIF case is equivalent to the 1LIF case with a short time lag (the rise time of the somatic voltage due to the plateau-conductance). This is because there is no synaptic barrage into the soma, so all synaptic integration occurs in the dendrite. The somatic voltage is thus guaranteed to reach threshold whenever the dendritic threshold is crossed.

In Figure 5, we observe how Δ_IE_ modulates the plateau time (the time when the dendrite reaches threshold, blue curves) as well as the spike time (red curves). In the direct case (Figure 5A), a small spike time modulation in the plateau time becomes increasingly noisy as inhibition becomes more in phase with excitation. Here, the dendrite acts as a 1LIF, so that the results are similar to those of Figure 2, where zero Δ_IE_ has high jitter (Figure 2F), and increasing Δ_IE_ decreases the mean spike time, although importantly at a slower rate (*ST*_Δ_ in Figure 2B compared to Figure 4B). Moreover, without somatic inhibition, the plateau potential can only transform plateau time into spike time by adding a fixed temporal offset (due to the fixed rise-time of the plateau-conductance). Because of this, the direct case features spike time modulation that is equivalent to plateau time modulation with a fixed added time.

In the gating case (Figure 5B), somatic inhibition does not influence the dendritic membrane potential because somato-dendritic interaction only occurs in one direction in our model. Therefore, the plateau time does not depend on Δ_IE_. The monotonically changing spike timing is attributed to somatic inhibition and spike timing can be modulated by more than 2.5 σ_in_ by temporal input offsets in the gating case, compared to about 1 σ_in_ spike time modulation in the direct case. Additionally, we find that the jitter remains within 0.3 σ_in_ in the gating case, while ranging from 0.3 to 0.6 σ in the direct case.

How well can a neuron simultaneously control mean spike time and reduce jitter? To answer this question we plotted jitter as a function of *ST*_Δ_ for all simulations conducted in the 1LIF (equivalent to direct inhibition) and 2LIF (equivalent to gating inhibition) cases (Figure 5C). For direct inhibition, where the postsynaptic potentials of both excitatory and inhibitory inputs directly interact in a single compartment, jitter increases linearly as *ST*_Δ_ increases. Thus, finding a point in parameter space where spike time is modulated with little noise is difficult. Alternatively, for gating inhibition, where excitatory and inhibitory barrages are separated into distinct spatial compartments, a large portion of parameter space simultaneously modulates spike timing by temporal input offset with attenuated noise.

## Discussion

In the first part of our study, we explore the response of conventional LIF units to barrages of inputs. A previous study found that barrages of inhibition increase jitter in LIF units due to an increase in degrees of freedom (Marsalek et al., 1997). Since the time of that computational work, experiments looking at the temporal offset of different current sinks and sources have given credence to the idea that different barrages of inputs can arrive separated by certain intervals of time (Klausberger and Somogyi, 2008; Mizuseki et al., 2009). Thus, in this study we included temporal input offsets and find that increases in jitter are tempered if inhibitory barrages are sufficiently offset in time from excitatory barrages (Figure 2). We further find a tradeoff between control of spike timing by temporal input offset and the ability to decrease spike jitter (Figure 5C, blue). In other words, although jitter can be reduced by offsetting inhibition from excitation, the ability of inhibition to shift spike timing is reduced the more temporally offset synaptic barrages become, in the case where all synaptic input occurs in a single compartment.

Significant synaptic and membrane noise is a relevant phenomenon *in vivo* (Pare et al., 1998) rendering the possibility of synaptic fine-tuning (e.g., to balance jitter and temporal control of spikes) remote. Additionally, pyramidal neurons do not act as isopotential units. Experiments reveal the existence of a Ca-hotzone in the apical dendrites, as well as voltage dependent sodium channels and NMDA receptors that can induce long-lasting plateau potentials and cause somatic depolarizations (Schiller et al., 1997; Seamans et al., 1997; Larkum et al., 1999, 2009; Milojkovic et al., 2007; Antic et al., 2010). We modeled the effects of these dendritic spikes on spike time control first in a biophysical two-compartment (Figure 3) model and, then, in an abstracted two-component LIF model (2LIF, Figure 4), and found that a spatial separation of inhibitory and excitatory barrages into a single neuron that supports dendritic plateau potentials can manipulate spike timing while reducing jitter (Figure 5).

The 2LIF model proposed herein lies between simple 1LIF models and more biophysically realistic multicompartmental models (Koch, 1999; Poirazi et al., 2003a; Hay et al., 2011) by keeping the parameter space limited while preserving important biophysical realities such as plateau potentials and spatially segregated synaptic input. In the *somatic-unit* of the 2LIF, a depolarizing plateau-conductance is activated whenever the *dendritic-unit* reaches threshold. Dendritic excitation activates the plateau-conductance in the soma, which, in turn, can be gated by somatic inhibition leading to precise spike timing (Figures 4, 5). Such a mechanism depends on the spatial separation of synaptic inputs (Pouille and Scanziani, 2001; Palmer et al., 2012) as well as their communication via the plateau-conductance (Figure 5). Here we argue that dendritic non-linearities confer distinct functionalities to dendritic and somatic input with respect to the somatic action potential. Because this causes the input-output function of neurons to be a mapping from an (at least) two-dimensional input space to a one-dimensional output space, it would be difficult to reproduce the results shown here in a 1LIF model that lacks additional degrees of freedom for the input.

Several multi-component models have been proposed as abstractions of pyramidal neurons to account for properties of dendrites. The sigma-pi unit features groups of inputs that multiply before being summed by weight and have been used to model spatio-temporal clustering needed for local NMDA-spike generation in dendrites (Mel, 1992b). The *clusteron* model (Mel, 1992a) features a spatial window of supralinearity, allowing for continuous spatio-temporal clustering effects in the dendrites. Similarly, single-neuron computation has been represented by two-layer neural networks (Poirazi et al., 2003b) allowing individual dendritic branches to act as a first layer of independent computation whose outputs are fed into the threshold operation at the soma. Here we aim to model the effect of dendritic plateau potentials, and the effect of the resultant plateau potentials on somatic spiking.

It has been proposed that multiple and independent computations (often NMDA-mediated) can provide input to the Ca-hotzone (Polsky et al., 2004). Extensions to the 2LIF might be needed to capture more detailed or complicated single-neuron functionalities. For instance, extra “NMDA-compartments” can be added which feed into the dendritic compartment. These extensions, as well as other details like morphology, spines, plasticity, adaptation, backpropagation, etc. could be added at will to increase the level of detail [for a review on how active dendritic conductance relate to these concepts see (Papoutsi et al., 2014)]. Here we provide a simple conceptual model that accounts for the basic non-linear layout of ion channels in a cell.

There are a number of two-compartment models with several ionic conductances that focus on interactions between soma and dendrites (Pinsky and Rinzel, 1994; Mainen and Sejnowski, 1996; Larkum et al., 2004; Murayama et al., 2009; Jadi et al., 2012). Here, we feature a non-traditional two-compartment model (2LIF), since the somatic and dendritic compartments are not “coupled” by a linear resistance but instead by a rectifying diode (the plateau conductance). Our 2LIF model has no explicitly simulated ionic conductances except a passive leak and the plateau-conductance, greatly decreasing the number of parameters needed to define our cell. Additionally, the plateau-event in our model is abstracted as a stereotyped, voltage-independent conductance change in the soma, as opposed to a voltage-dependent conductance change in the dendrites. Thus, our model is designed to be as simple as possible while still allowing for the range of behaviors relevant to spike timing control.

In particular, we compared two types of spike timing control mechanisms, one by changing the temporal input offset (*ST*_Δ_), and the other by changing the inhibitory strength (*ST*_inh_). We found that *ST*_inh_ was associated with a tradeoff between controlling spike time and reducing jitter. Though this tradeoff existed in both the 1LIF and 2LIF cases, it was largely tempered in the 2LIF case (compare Figure 2C to Figure 4C and Figure 2F to Figure 4F). In both cases, the no temporal input offset cases were especially poor at controlling spike timing and reducing output jitter simultaneously. The difference between the 1LIF and 2LIF case was especially stark in *ST*_Δ_. In 95.9% of the parameter space tested, *ST*_Δ_values were higher in the 2LIF than in the 1LIF, and in the majority of parameter space the 2LIF *ST*_Δ_ values were at least 3 times higher than in the 1LIF.

We focused on the timing of the first action potential, neglecting other aspects of neural coding such as frequency modulation. We did so chiefly because such spike timing is particularly relevant to areas of the brain like the hippocampus, where robust relationships between spatial location and spike timing exist (O'Keefe and Recce, 1993). Additionally, despite *in vitro* evidence that dendritic electrogenesis contributes to frequency control in pyramidal neurons, our model lacks the complexity to capture such effects. These are highly non-linear, resulting from a number of non-linear voltage dependant currents acting in concert (Williams and Stuart, 1999; Su et al., 2001; Metz et al., 2007). Instead of instantiating these complexities, we focused on making the model as simple as possible and relegated ourselves to studying only spike timing.

Our findings have important ramifications for the temporal encoding of neurons in the presence of barrage-like synaptic input. For example, the “dual-oscillator interference model” (Burgess et al., 2007) used to explain phase precession in entorhinal cortex grid cells features spatial segregation of inputs into the dendrites and soma. The inputs are of slightly different frequencies, causing an interference pattern and a modulation of spike phase. We likewise observe monotonically increasing spike timing modulation in our 2LIF model by changing the input offset (phase) of the inhibitory barrage relative to the excitatory barrage (Figure 5B).

Of particular note is a study looking at the relationship between local dendritic and global neuronal processing in dual-oscillator interference models, considering the realistic electrogenic structure of stellate cells (Remme et al., 2010). In that study, an important tradeoff was found between local and global processing as a function of the coupling between the different compartments of the cell. Any dual-oscillator interference model needs both independent oscillations to exist in each compartment (local processing) as well as their interaction in order to create interference (global processing). If, instead, the electrotonic structure is such that multiple compartments can phase lock, then local oscillations cannot independently exist and phase precession cannot be realized. While stellate cells have a soma with a single group of dendrites radiating outward from it, pyramidal neurons have an elongated apical trunk that bifurcates into a second group of thin dendritic tufts. The apical trunk hosts a number of non-linear channels. Relevant to this discussion are the voltage-dependent sodium channels that only turn on for propagating suprathreshold signals, and the HCN channels, which act to lengthen the electrotonic distance of the neuron, especially with respect to subthreshold events. Finally, pyramidal neurons uniquely possess the calcium “hot-zone” at the apical bifurcation that supports the long-lasting calcium spike. Taken together, these facts point to a compartmentalization of the neuron into independent compartments that can interact exclusively with suprathreshold events. Thus, in our study, both local and global processing can coexist in the same neuron. Our result is wholly dependent on the subthreshold independence of the two compartments that only interact via suprathreshold signaling.

In our model we considered exclusively the dendrite-to-soma propagation of the calcium spike, and not the back propagation of action potentials. This assumption is valid in regimes where somatic spiking is largely induced by apical dendrite input, and not basal dendrite input. Indeed, axons from Schaffer collaterals and the perforant pathway both send strong inputs into the apical dendrites of pyramidal neurons in the hippocampus (Jarsky et al., 2005). In more recent *in vivo* work, it has been shown that apical dendritic signals correlate much better to action potential output than do signals in the closer basal dendrites in pyramidal neurons (Palmer et al., 2014).

Moreover, it is known that theta-locked entorhinal cortex spiking co-exists in time with a current sink in their targets, *stratum lacunosum moleculare* in CA1, where the apical tuft of CA1 pyramidal neurons lie. These cells spike between 90° and 180° of theta (i.e., 50–150 ms) later (Mizuseki et al., 2009). Such a lag, hypothesized to provide temporal windows for local circuit computation (Buzsaki, 2010), is inconsistent with passive integration of synaptic inputs. Our results support the notion that in addition to the possibility of local circuit computation, the spatiotemporal distribution of spiking, and current sources/sinks during navigation in the entorhinal/hippocampal circuit (in particular the lag between synaptic input and postsynaptic firing in CA1 cells) and the distribution of membrane channels that support plateau potentials in pyramidal neurons can be explained by a mechanism where dendritic inputs cause a sustained depolarization in the soma that can be manipulated via somatic inhibition over longer timespans.

Computational network models and some theoretical work have suggested that perisomatic and dendritic inhibition have distinct roles in the generation of spikes during sharp wave ripples in the hippocampus (Cutsuridis and Taxidis, 2013; Taxidis et al., 2013). Experimental work by Royer et al. (2012) has explored these distinct effects in the CA1 pyramidal neurons during navigation. Upon suppression of soma-targeting, but not dendrite-targeting, interneurons, the range of phase precession was reduced during navigation by more than a factor of 2 (Royer et al., 2012 their Figure 6). Similarly, in the 2LIF model, a decrease in somatic inhibitory strength has a detrimental effect on spike timing control, since the ability to gate the plateau-conductance decreases (Figures 4, 5). Importantly, manipulating dendritic inhibition in our model does not drastically curtail the cell's phase modulation, since the majority of the spike timing control is due to gating of the plateau-conductance by somatic inhibition, in agreement with findings of Royer and colleagues.

In our study, input distributions were fixed at an input jitter of 40 ms, and thus the results may only be relevant for brain states supporting inputs with similar temporal characteristics. The 40 ms input jitter used here is comparable to the distribution of presynaptic cell firing in entorhinal cortex that project to the hippocampus (Mizuseki et al., 2009). In general, the parameter space of the input is large and multidimensional, involving the numbers of synaptic events, their strengths, temporal distribution shape and frequency, and the relative amount of excitation and inhibition. We neglected to search this space exhaustively and instead chose parameters with physiological relevance to sustained depolarizations in the cortex. However, due to the ubiquity of the dual integration zone feature in neurons throughout the brain, a model such as ours presented here may describe computation in other brain areas, with synaptic noise of different temporal characteristics, or where temporal coding is not the predominant mode of computation.

### Conflict of interest statement

The authors declare that the research was conducted in the absence of any commercial or financial relationships that could be construed as a potential conflict of interest.
