# Supplementary material for: Spike-timing control by dendritic plateau potentials in the presence of synaptic barrages
Source: Front Comput Neurosci. 2014 Aug 14;8:89. doi: 10.3389/fncom.2014.00089 (PMC4132263; doi:10.3389/fncom.2014.00089)
Supplement: Supplementary file 1 [file DataSheet1.DOCX]

Supplemental Material

1 Figure


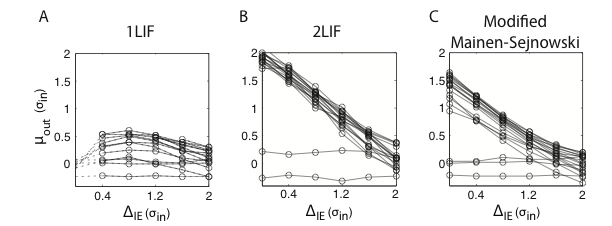


**Supplementary Figure 1.** Mean spike time changes linearly as a function of temporal input offset in the 1LIF (A), 2LIF (B), and Modified Mainen-Sejnowski model (C). Plotted are the mean spike times (circles) for each of 6 input offsets (x-axis), for 25 different choices of excitation and inhibition strength evenly spaced across the entirety of those tested. In the 1LIF case (A), the 0 input offset values were not included since they did not follow the linear trend and would have underestimated *ST*_∆_ values, but are shown here with dotted lines. Filled lines directly connect mean spike times of the nearest input offsets tested, obtained from simulations with the same excitatory and inhibitory strength values (these are not the best fit lines).
